# Hand, foot, and mouth disease: could EPs® 7630 be a treatment option? A prospective randomized open-label multicenter clinical study

**DOI:** 10.3389/fped.2024.1274010

**Published:** 2024-05-20

**Authors:** Murat Sütçü, Manolya Kara, Funda Yıldız, Ömer Kılıç, Tugce Tural Kara, Gulsen Akkoc, Ayşe Büyükçam, Şefika Elmas Bozdemir, Özlem Özgür Gündeşlioğlu, Doruk Gül, Merve İseri Nepesov, Ateş Kara

**Affiliations:** ^1^ Pediatric Infectious Diseases, Faculty of Medicine, Istinye University, Istanbul, Türkiye; ^2^ Pediatrics, Faculty of Medicine, Istinye University, Istanbul, Türkiye; ^3^ Pediatric Infectious Diseases, Faculty of Medicine, Yeditepe University, Istanbul, Türkiye; ^4^ Pediatric Infectious Diseases, Faculty of Medicine, Eskisehir Osmangazi University, Eskisehir, Türkiye; ^5^ Pediatric Infectious Diseases, Faculty of Medicine, Akdeniz University, Antalya, Türkiye; ^6^ Pediatric Infectious Diseases, University of Health Sciences Van Research and Training Hospital, Van, Türkiye; ^7^ Pediatric Infectious Diseases, Cengiz Gokcek Maternity and Children's Hospital, Gaziantep, Türkiye; ^8^ Pediatric Infectious Diseases, Bursa Dortcelik Children's Hospital, Bursa, Türkiye; ^9^ Pediatric Infectious Diseases, Faculty of Medicine, Çukurova University, Adana, Türkiye; ^10^ Pediatric Infectious Diseases, Faculty of Medicine, Hacettepe University, Ankara, Türkiye

**Keywords:** hand, foot, mouth disease, EPs® 7630, children, herbal remedy, clinical study

## Abstract

**Purpose:**

Hand, foot and mouth disease (HFMD) is a viral contagious disease of children caused by human enteroviruses (EVs) and coxsackieviruses (CVs). There is no specific treatment option for HFMD. EPs® 7630's anti-infective and immunomodulatory properties have previously been demonstrated in several *in vitro* and *in vivo* studies; however, the use of this herbal medicine in children with HFMD has not previously been investigated.

**Methods:**

This prospective randomized multicenter clinical study included 208 children with HFMD. The diagnosis was made by pediatricians. The patients who were within the first 48 h of symptom onset (according to the first onset of fever and skin findings) were enrolled. The study participants were assigned into 2 groups as EPs® 7630 and control groups. All patients were followed up twice more, 48 h after the first admission and on the 5th–7th day. Another phone evaluation was conducted for those with continued complaints from the previous visit.

**Results:**

The median age was 27 (12–112) months. The male-female ratio was 0.98. One hundred thirty one (63%) of 190 patients had no history of household contact. EPs® 7630 group included 94 and control group included 96 patients. A significant difference was found between the groups in terms of complaint scores at the visits made at the 48th h of the treatment and on days 5–7 (*p* < 0.001). The mean ± SD disease duration of EPs® 7630 users was significantly shorter 6.07 ± 0.70 days (95% CI: 5.92–6.21)] than the control group [8.58 ± 0.94 days (95% CI: 8.39–8.77)] (*p* < 0.001). Besides, the hospitalization rate among the EPs® 7630 users were significantly lower (*p* = 0.019). No side effects were observed, except for unpleasant taste, which was reported in 5 patients (EPs® 7630 group).

**Conclusion:**

Considering its efficacy and safety profile EPs® 7630 may represent a feasible herbal-based treatment option for children with HFMD.

**Clinical Trial Registration:**

ClinicalTrials.gov, identifier (NCT06353477).

## Introduction

Hand, foot, and mouth disease (HFMD) is a common viral contagious disease of children (1). It is caused by picornaviruses, specifically human enteroviruses (EVs) and coxsackieviruses (CVs) (2). Although CV-A16 and EV-A71 were the most frequently responsible serotypes for a long time, CV-A6 and CV-A10 have been linked to various outbreaks in Asia, America, and Europe in recent years (3–6). The disease is characterized by fever, painful oral enanthem, and a macular, maculopapular, or vesicular rash on the hands and feet (1, 7, 8). The most common complication is decreased oral intake, which can lead to dehydration and may require hospitalization in young infants (9, 10). The treatment of HFMD is mainly supportive, and no specific antiviral therapy is available. Nonetheless, when assessed in terms of clinical safety and accessibility, herbal formulations with antiviral and/or immunomodulatory activity can be employed (4).

EPs® 7630 is a proprietary extract and active ingredient from the roots of *Pelargonium sidoides*. It is effective for the treatment of several common respiratory tract infections (RTIs) (11, 12). The antiviral, antibacterial, and immunomodulatory properties of EPs®7630 have previously been reported (13–17). Although EPs® 7630's mode of action is not fully understood, its antiviral activity has been linked to several pathways, including virus interference with host cell receptors, inhibition of viral replication, inhibition of cytopathic effect, and modulation of interferon (IFN) system (14, 16, 17). EPs® 7630 has been shown to have effects mostly against enveloped viruses, including respiratory syncytial virus (RSV), parainfluenza virus (PIV), influenza A virus (IV; H1N1, H3N2), and human coronavirus (HCoV-229E). Even so, it has also been demonstrated that EPs® 7630 inhibits the non-enveloped virus CV (16).

To the best of our knowledge, there is no published research on the treatment of EPs® 7630 during HFMD. This randomized controlled study aims to evaluate the effectiveness and safety of the pharmaceutical extract EPs® 7630 from *P.sidoides* in treating hand, foot, and mouth disease in children. The study will investigate the impact of EPs® 7630 on the severity of the disease over a specific period and its effects on hospitalization rates and potential complications. This research aims to contribute to the treatment of hand, foot, and mouth disease in children.

## Material and methods

### Study design

This multicenter randomized controlled study was conducted between June 2019 and June 2022 in 8 centers in Turkey. These centers were hospitals of reference that provided tertiary care services. The clinical study protocol was approved by the Eskisehir Osmangazi University. This clinical study protocol was approved by the Eskisehir Osmangazi University Interventional Research Ethics Committee with the number 2019–2010 and conducted in accordance with the World Medical Association's Declaration of Helsinki and on Good Clinical Practice compliance. The study is registered at ClinicalTrials.gov (NCT06353477).

Written informed consent was obtained from parents of all patients included in the study.

### Study participants and clinical management

All of pediatric patients who were examined by a pediatrician and diagnosed with HFMD and start of the symptoms in last 48 h (either fever or enanthems or exanthem) were offered trial participation. Patients whose complaints lasted more than 48 h, those whose families stated that they were unable to comply with follow-ups, those did not give informed consent, those taking another antiviral or supportive treatment, those who had used antibiotics in the previous 1 month, those with a history of immunodeficiency or a family history of immunodeficiency, and those with a previous history of anaphylaxis with any supplement or drug, any chronic disease, or skin lesion were not included in the study.

At the first admission, the duration of the patients’ complaints, the distribution of the lesions in the body, and the fever status were recorded. Parents were asked to rate the severity of the child's restlessness, inappetence, and sleeplessness status on a scale of 0–10.

Participants were assigned 1:1 to one of two trial arms by a local research team member using a centralized computerized randomization system (RAND2 software, The MathWorks Inc, Natick, United States, contractually managed by the data management team). Lists in four blocks were added to the automatic online randomization system to ensure a homogeneous distribution of the groups in both study centers. On the basis of the power calculations of similar studies, a minimum sample size of 80 per group was calculated to give a 90% probability (power) of producing a significant finding. Overall, 120 patients were designated for the for each group that considering that there may be losses in the study.

All patients were followed up twice more, 48 h after the first admission and on the 5th–7th days. Another phone evaluation was conducted for those with continued complaints from the previous visit. During these visits, the patient's fever status, restlessness, inappetence, and sleeplessness scores were asked again of their parents and recorded. Patient medication adherence and drug side effects were evaluated. After the patient's recovery, the total duration of the disease and the duration of restlessness, inappetence, and sleeplessness were recorded. Patients who were hospitalized or developed complications were noted.

### Intervention

EPs® 7630 is an extract from the roots of *Pelargonium sidoides*, drug-extract ratio 1:8–10, extraction solvent ethanol 11% (w/w). The patients were divided into two groups: (i) group 1 received herbal drug EPs® 7630 by oral route [Umca® solution; (3 × 10 drops; between 1 and 5 years of age, 3 × 20 drops; 6–12 years of age, 3 × 30 drops for children >12 years of age)] for 7 days and (ii) group 2 (control group) did not receive any herbal medication. The medication was administered orally, at least 30 min before or after meals. Patients in both groups were prescribed paracetamol (10 mg/kg/dose, 4 times a day, maximum 4,000 mg/day.) as an antipyretic agent. Temperature measurement was made at home and in the hospital via the axillary route.

### Statistical method

Analyses were performed by a prospectively defined analysis plan. Using the SPSS v28.0 (Statistical Product and Service Solutions, IBM). Categorical variables were presented as numbers (*n*) and percentages (%). The variables were investigated using the Kolmogorov–Simirnov test to determine whether or not they are normally distributed. Descriptive analyses were presented using medians and interquartile range (IQR) for the non-normally distributed variables. Kruskal–Wallis tests were conducted to compare these variables. Categorical data were compared by the *χ*^2^ test and Fisher exact test. For variables not distributed normally, the Mann–Whitney *U*-test was used. The *p*-value of less than 0.05 was considered to show a statistically significant result.

## Results

**Figure 1:**
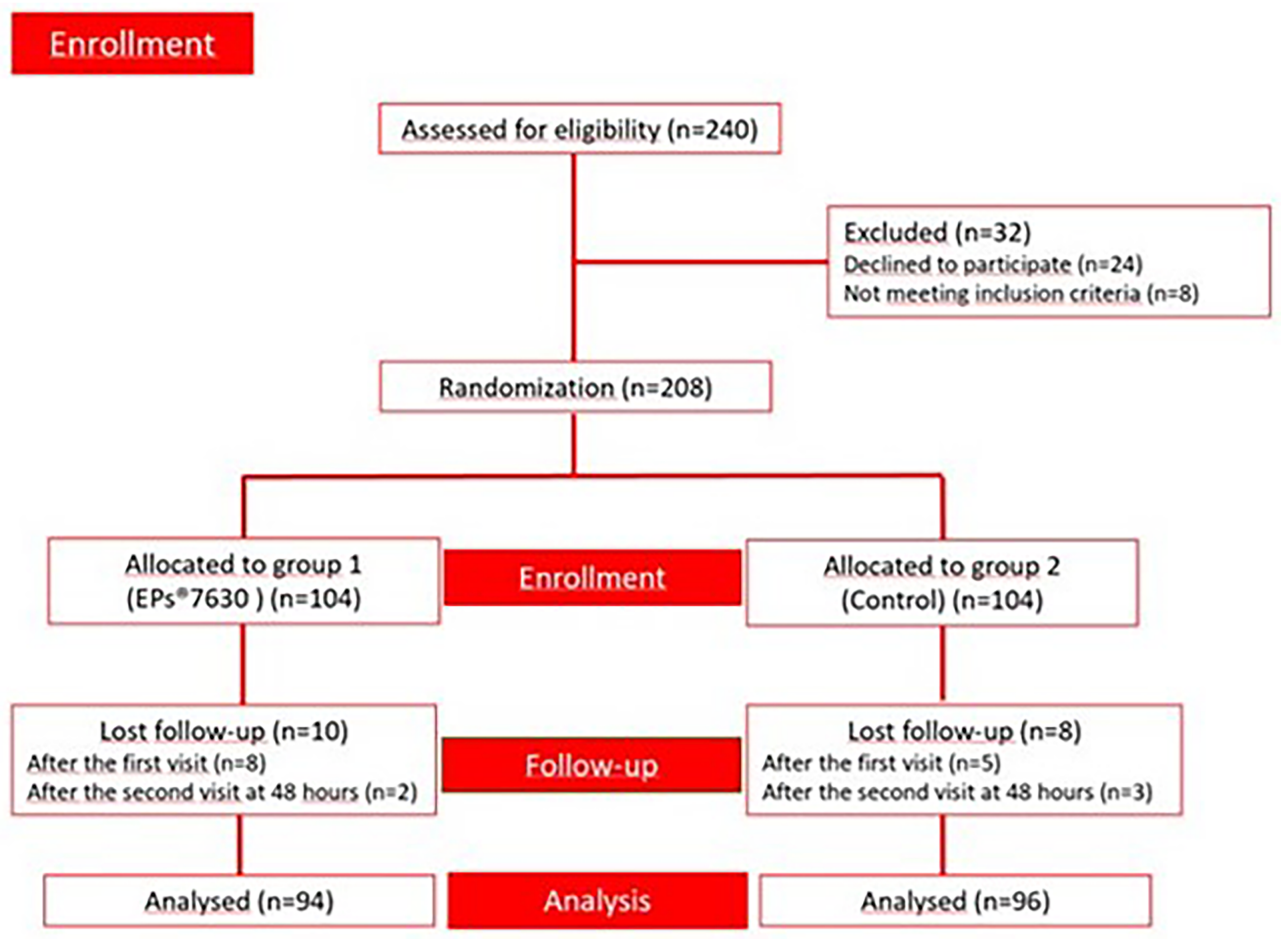
Enrolment chart of the study.

After the initial assessment, 32 children were excluded [declined to participate (*n* = 24) and did not meet inclusion criteria (*n* = 8)]. A total of 208 children diagnosed with HFMD were included in the study. During the study period, a total of 18 patients [EPs® 7630 (*n* = 10); control group (*n* = 8)] were lost follow-up and excluded from the final analysis. Then, 190 children [EPs® 7630 (*n* = 94); control group (*n* = 96)] were analysed (Figure 1).

### Clinical characteristics

The median age was 27 (12–112) months. The male-female ratio was 0.98. One hundred thirty one (63%) of 208 patients had no history of household contact (Table 1). The number of patients with sibling contact history was 28 (13.5%) and the cousin contact was 35 (16.8%). Furthermore, 14 (6.7%) patients had contact with other people. The onset of symptoms in 138 patients (66.3%) was one day ago, 70 (33.7%) patients two days ago when they were admitted to the hospital.

**Table 1:** Baseline results of the patients.

Parameters (*N*)	All patients (208)	EPs® 7630 (104)	Control (104)
Age (month, median, range)	27 (12–112)	26 (12–110)	28 (12–112)
Sex, female (*n*, %)	103 (49.5)	52 (50)	51 (49)
Pre-admission complaint time (day, median, range)	1 (1–2)	1 (1–2)	1 (1–2)
Household contact *n* (%)	77 (37)	39 (37.5)	38 (36.5)
The distribution of intraoral lesions (*n*, %)			
Lips	63 (30.3)	29 (27.9)	34 (32.7)
Tongue	89 (42.8)	50 (48)	39 (37.5)
Cheeks	57 (27.4)	31 (29.8)	26 (24.1)
Palatine	175 (84.1)	84 (80.8)	91 (87.5)
The distribution of rash (*n*, %)			
Only extremities	73 (35.1)	34 (32.7)	39 (37.5)
Trunk and extremities	76 (36.5)	38 (36.5)	38 (36.5)
Whole body	61 (29.3)	33 (31.7)	28 (26.9)
Presence of fever (*n*, %)			
<37.5	53 (25.5)	17 (16.3)	36 (34.6)
37.5–38.5	101 (48.6)	58 (55.7)	43 (41.3)
≥38.5	54 (25.9)	29 (27.9)	25 (24)

A total of 193 (92.8%) patients had intraoral [palate (*n* = 175, 84.1%), tongue (*n* = 89; 42.8%), cheek (*n* = 57, 27.4%), lip (*n* = 63; 30.3%)] lesions. While 73 (35.1%) patients had rashes only on the extremities, 76 (36.5%) had rashes on the trunk and extremities. Additionally, 61 (29.3%) patients had rashes in the whole body.

### Comparison of the two groups

EPs® 7630 was prescribed to 94 (49.5%) patients whereas 96 (50.5%) patients were determined as the control group. While 127 (66.78%) patients were not prescribed any other medication, 63 (33.2%) were prescribed paracetamol (Table 2). No statistically significant difference was observed in terms of paracetamol use between the two groups [EPs® 7630 (*n* = 27, 28.7%), control (*n* = 36, 37.5%), *p*-value = 0.19].

**Table 2:** Treatment results of the patients whose all visits are completed.

Parameters	All patients	EPs® 7630	Control
Treatment (*n*, %)	190 (100)	94 (49.5)	96 (50.5)
Additional drugs (*n*, %)			
Paracetamol only	52 (27.4)	22 (23.4)	30 (31.3)
Antihistaminics only	4 (2.1)	1 (1.1)	3 (3.1)
Paracetamol & antihistaminics	11 (5.8)	5 (5.3)	6 (6.2)
Hospitalization (*n*, %)	9 (4.7)	1 (1.1)	8 (8.3)
Reason for hospitalization			
Inability to feed	2 (1.1)	1 (1.1)	1 (1.1)
Prolonged fever	4 (2.1)	–	4 (4.2)
Secondary bacterial skin infection	3 (1.6)	–	3 (3.1)
Length of hospital stay (day, median, range)	2 (2–4)	3	2 (2–4)
Total duration of symptoms after treatment (day, median, range)			
Fever	2 (0–6)	2 (1–5)	4 (0–6)
Irritability	6 (3–10)	5 (3–7)	8 (6–10)
Decrease of apetite	7 (3–10)	5 (3–7)	7 (5–10)
Sleeplessness	6 (4–10)	6 (4–8)	7 (5–10)
Duration of illness (day, mean ± SD)	7.34 ± 1.5	6.07 ± 0.70	8.58 ± 0.94

The median age of the group prescribed EPs® 7630 was 26 months (12–110 months) and the median age of the control group was 28 months (12–112 months). There was no statistically significant difference between the two groups (*p* = 0.502).

**Figure 2:**
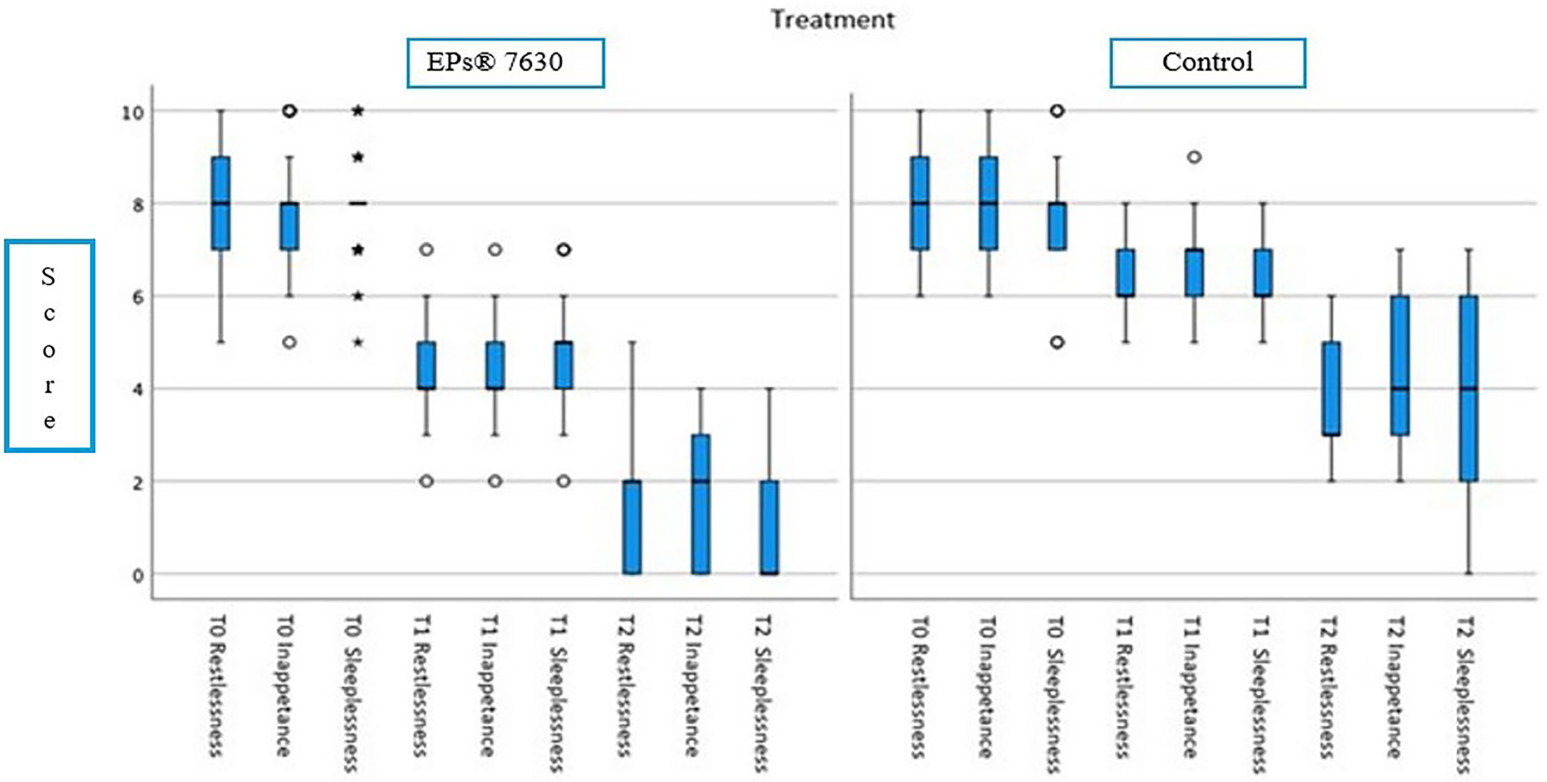
Median complaint scores of the groups before treatment (T0), at 48 h of treatment (T1), and at 5–7 days of treatment (T2).

At the beginning of the treatment, 17 (16.5%) of the patients given EPs® 7630 had a body temperature below 37.5°C, 58 (55.7%) patients between 37.5°C and 38.5°C and 29 (27.9%) patients over 38.5°C. In the control group, 36 (34.6%) had a body temperature below 37.5°C, 43 (41.3%) patients between 37.5°C and 38.5°C, and 25 (24%) patients over 38.5°C. There was no significant difference between the EPs® 7630 and control groups in the scores of restlessness, inappetence, and sleeplessness at the time of admission to the hospital. However, a significant difference was found between the groups in terms of complaint scores at the visits made at the 48th h of the treatment and on days 5–7 (*p* < 0.001) (Table 3 and Figure 2).

**Table 3:** Median (25%–75%) complaint scores measured before treatment, at 48 h of treatment, and at days 5–7 of treatment among EPs® 7630 and control groups.

	EPs® 7630 Median (25%–75%)	Control Median (25%–75%)	*P*-value
Before treatment			
Restlessness	8 (7–9)	8 (7–9)	0.870
Inappetence	8 (7–8)	8 (7–9)	0.124
Sleeplessness	8 (7–8)	8 (7–8)	0.514
At 48 h of treatment			
Restlessness	4 (4–5)	6 (6–7)	**<0**.**001**
Inappetence	4 (4–5)	7 (6–7)	**<0**.**001**
Sleeplessness	5 (4–5)	6 (6–7)	**<0**.**001**
At days 5–7 of treatment			
Restlessness	2 (0–2)	3 (3–5)	**<0**.**001**
Inappetence	2 (0–3)	4 (3–6)	**<0**.**001**
Sleeplessness	0 (0–2)	4 (2–6)	**<0**.**001**

Bold values are statistically significant.

Nine patients (4.7%) were hospitalized with the median duration of 2 (2–4) days. [EPs® 7630 (*n* = 1) and control (*n* = 8)], The hospitalization rate among the EPs® 7630 users were significantly lower (*p* = 0.019). The median age of the patients who were admitted to the hospital was 33 months (15–90 months). Out of a total of 9 patients, 6 were male. Among them, 5 patients had a fever above 38.5°C. Four patients were admitted to the hospital due to prolonged fever. A rash was observed all over the body in 6 out of 9 patients. Three patients had a secondary bacterial skin infection. Eight intraoral lesions were detected in the patients. Two patients were hospitalized because they were unable to take oral intake or feed.

**Figure 3:**
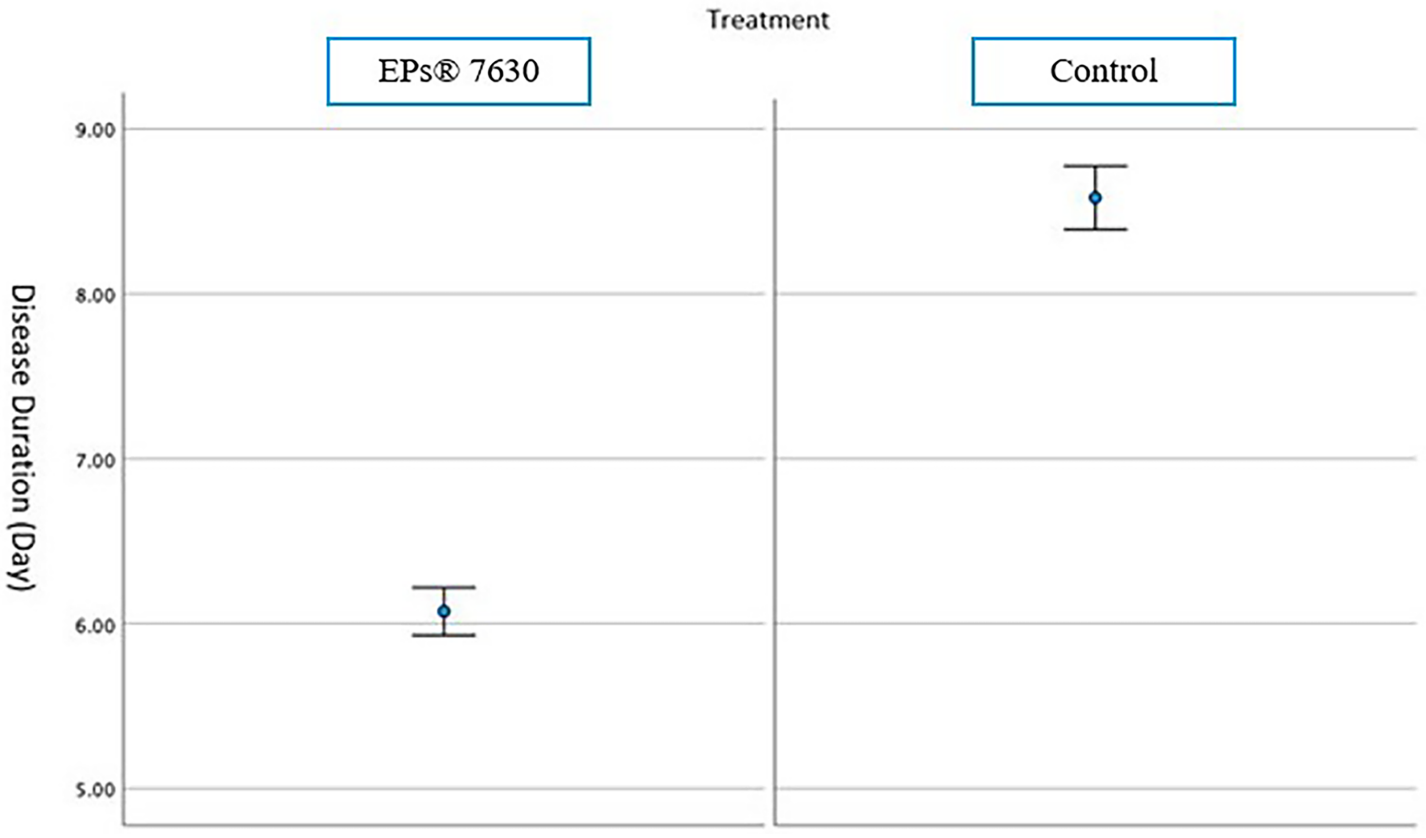
Mean and 95% CI disease duration of EP7630 and control group.

The mean ± SD disease duration of EPs® 7630 users was 6.07 ± 0.70 days (95% CI: 5.92–6.21) and the mean ± SD disease duration in the control group was 8.58 ± 0.94 days (95% CI: 8.39–8.77). A statistically significant difference was found between the two groups in terms of the duration of the disease (*p* < 0.001) (Figure 3).

No side effects were observed, except for unpleasant taste, which was reported in 5 patients in EPs® 7630 group.

## Discussion

EPs® 7630 has antiviral and antibacterial activity and immunomodulatory properties (13–17). However, knowledge of EPs® 7630's exact action mechanism is still limited. Literature data indicate that the compound provides an effect at more than one point in the infectious cascade, and the interactions are complex. According to the *in vitro* studies, the antibacterial activity of EPs® 7630 comes from an immune modulation-mediated pathway that leads to macrophage activation (via the cytokine interferon-gamma; IFN-ɣ) and an increase in nitric oxide production in response (15, 18). Other effects of EPs® 7630 include the production of cytokines (such as IL-1, IL-2, and tumor necrosis factor-α), the modulation of secretory immunoglobulin A in saliva, the production of IL-15 in serum and nasal mucosa, and the secretion of IL-6 in the serum (13, 15).

EPs® 7630's significant antiviral activity has also been linked to several pathways, including virus interference with host cell receptors, inhibition of viral replication, inhibition of cytopathic effect, and modulation of IFN system (14, 16, 17, 19). In a report by Michaelis et al. (16), EPs® 7630 decreased infectious titers of all susceptible viruses, including CV, dose-dependently. Another exciting research reveals that upregulating the vitamin D receptor and epithelial cell differentiation helps to improve host defense against rhinovirus infection (20).

The efficacy of EPs® 7630 in various acute RTIs was evaluated both in children and adults (11, 12). According to a systematic review and meta-analysis, EPs® 7630 is both safe and effective at treating a range of conditions. These include acute bronchitis in children and adults, acute rhinosinusitis in adult patients, and acute tonsillopharyngitis in children. Patients who were treated with this herbal extract experienced a rapid onset of remission, fewer and less severe symptoms, and were able to return to work, school, or kindergarten sooner than those who received a placebo (12). Clinical studies also revealed that the use of EPs® 7630 significantly reduced the severity of symptoms and shortened the duration of common cold in adult patients (21–23). In Seifert's et al. (24) report, EPs® 7630 relieved symptoms, accelerated recovery, and, in addition reduced paracetamol use in children with RTIs. To date, there is no clinical study conducted previously on the use of *P.sidoides* for treating HFMD. In the present prospective, multicenter research, EPs® 7630 use in this patient group was significantly superior to control in terms of complaint scores of restlessness, inappetence, and sleeplessness at the visits made both 48th h and 5–7 days of treatment. Similar to Seifert's et al paracetamol use was lower in EPs® 7630 group in this study, however the difference was not statistically significant.

HFMD can spread rapidly and cause outbreaks in kindergartens and schools due to several factors, such as close contact with children, not paying attention to hygiene rules, and long virus spread (6). The highest contagious period is in the first week of intense viral replication (25). The disease can also be transmitted to adults. The household transmission rate was previously found to be as high as 52%–84% (25). In the present study, the history of household contact was 35.3%, and 12.1% were siblings. However, this is the incidence data at the time of diagnosis, and we cannot comment on the transmission ratio since we did not perform case tracking afterward.

There is no specific antiviral therapy for HFMD. Symptomatic relief can be achieved with antipyretic and antihistamine drugs (6). Young infants may require intravenous fluids since feeding difficulties and dehydration may emerge due to painful intraoral enanthems. Likewise, 179 (94.2%) patients in our study cohort had intraoral lesions. As a result, pain related restlessness and inappetence were high.

In uncomplicated cases, the disease duration ranges between seven to ten days (26). In our study cohort, the mean disease duration of EPs® 7630 users was significantly shorter than the control group. Besides, despite the lower number of inpatients, the hospitalization rate was lower in the EPs® 7630 group. It would undoubtedly enhance the power of the study if it were blinded and had a control group that received a placebo. However, the study's finding is still valuable since computer-based patient randomization was performed instead of clinician selection.

EPs® 7630 has been shown to be most effective in the early stages of virus replication and cell attachment (16, 17, 27). In the present study, 67.4% of patients were admitted on the first day of symptom onset, which may have resulted in a relatively short disease duration due to early treatment initiation. Another issue that may be the subject of further research here is whether it will have a protective effect on the disease when it is given after contact while the condition is still in the incubation period.

Potential adverse reactions related to EPs® 7630 use include mild gastrointestinal (GI) side effects (diarrhea, epigastric discomfort, nausea or vomiting, dysphagia), mild nasal and gingival bleeding, and allergic reactions (28). Although previous reports raised suspicion about this herbal medicine and possible hepatotoxicity, subsequent articles were published stating that hepatotoxicity was not related to *P. sidoides* (29, 30). Many other reports also highlighted the safety and tolerability of this herbal medicine, both in children and adults (13, 23, 24, 31). A review analyzing eight randomized controlled trials concluded that EPs® 7630 was safe and well tolerated in children, with the most common adverse effect being mild GI disturbance (13). Likewise, in the present study, no severe adverse reaction was observed. Only five patients complained about the taste of the medicine.

### Strengths and limitations of the study

The present study is valuable due to its prospective multicenter nature, as it is the first study in the literature to investigate the effectiveness of EPs® 7630 in children with HFMD. The limitations of the study were the open-label design and the lack of a control-treatment (because the funding for a placebo-controlled trial was not available). Further preclinical and clinical studies in this area will be more instructive for the use of EPs® 7630 in the treatment of HFMD.

## Conclusions

The use of EPs® 7630 significantly reduced the complaint scores in children with HFMD, and it was well tolerated. Considering its efficacy and safety profile, EPs® 7630 may represent a feasible herbal treatment option for children with HFMD. Future research is required to assess EPs 7630's effectiveness in comparison to traditional therapy approaches and examine its antiviral activities against enteroviruses.

## Data Availability

The datasets presented in this study can be found in online repositories. This data can be found here: https://figshare.com/s/db6189642afd29ca59d7.
